# Laser Capture and Deep Sequencing Reveals the Transcriptomic Programmes Regulating the Onset of Pancreas and Liver Differentiation in Human Embryos

**DOI:** 10.1016/j.stemcr.2017.09.018

**Published:** 2017-10-19

**Authors:** Rachel E. Jennings, Andrew A. Berry, David T. Gerrard, Stephen J. Wearne, James Strutt, Sarah Withey, Mariya Chhatriwala, Karen Piper Hanley, Ludovic Vallier, Nicoletta Bobola, Neil A. Hanley

**Affiliations:** 1 Division of Diabetes, Endocrinology & Gastroenterology, Faculty of Biology, Medicine & Health, AV Hill Building, University of Manchester, Oxford Road, Manchester M13 9PT, UK; 2 Endocrinology Department, Manchester University NHS Foundation Trust, Grafton Street, Manchester M13 9WU, UK; 3 Bioinformatics Group, Faculty of Biology, Medicine & Health, University of Manchester, Oxford Road, Manchester M13 9PT, UK; 4 Wellcome Trust Sanger Institute, Hinxton CB10 1SA, UK; 5 Wellcome Trust-Medical Research Council Stem Cell Institute, Anne McLaren Laboratory and Department of Surgery, University of Cambridge, Cambridge CB2 0SZ, UK; 6 Division of Dentistry, Faculty of Biology, Medicine & Health, University of Manchester, Oxford Road, Manchester M13 9PT, UK

**Keywords:** human, pancreas, liver, development, embryo, transcriptome, RNA sequencing, stem cell

## Abstract

To interrogate the alternative fates of pancreas and liver in the earliest stages of human organogenesis, we developed laser capture, RNA amplification, and computational analysis of deep sequencing. Pancreas-enriched gene expression was less conserved between human and mouse than for liver. The dorsal pancreatic bud was enriched for components of Notch, Wnt, BMP, and FGF signaling, almost all genes known to cause pancreatic agenesis or hypoplasia, and over 30 unexplored transcription factors. SOX9 and RORA were imputed as key regulators in pancreas compared with EP300, HNF4A, and FOXA family members in liver. Analyses implied that current *in vitro* human stem cell differentiation follows a dorsal rather than a ventral pancreatic program and pointed to additional factors for hepatic differentiation. In summary, we provide the transcriptional codes regulating the start of human liver and pancreas development to facilitate stem cell research and clinical interpretation without inter-species extrapolation.

## Highlights

• Transcriptomic signatures at the inception of human liver and pancreas development
• Limited conservation of pancreas-enriched gene expression between human and mouse
• Human PSC protocols imply a dorsal rather than a ventral pancreatic program
• New pancreatic transcription factors imputed by differential analysis

## Graphical Abstract

**Figure:**
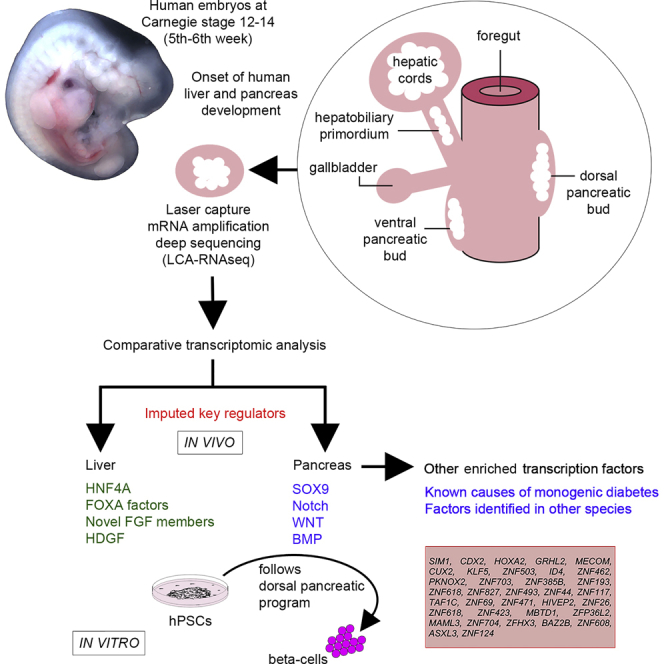

## Teaser

Jennings et al. present the first transcriptomes at the inception of liver and pancreas development in very early post-implantation human embryos. By computational analysis, they impute regulatory signatures in each organ, including new transcription factors in pancreas and new guidance for human pluripotent stem cell differentiation.

## Introduction

Experiments in mouse and other model organisms have demonstrated that the pancreas and liver arise as alternative fates from common progenitors in the distal foregut (McCracken and Wells, 2012). We know little about the earliest phases of their organogenesis directly in human embryos, the period correlating to organ agenesis or severe hypoplasia in patients. In major part this is due to the tiny size of the organ buds and the scarcity of post-implantation human embryonic material. Fresh understanding of human development has come from genome-wide deep sequencing (seq) technology. However, the earliest transcriptomic data for pancreas come 7–15 days after the bud first emerges from the distal foregut by which time the organ is branched with luminal structures, just prior to acinar differentiation, and in all likelihood comprised of heterogeneous progenitor cells (Cebola et al., 2015, Gerrard et al., 2016). By this stage, liver development has also advanced beyond the early bud. There are differences between the earliest human pancreas and its murine counterpart, such as lack of the transcription factor (TF), NKX2-2, and no early phase of endocrine differentiation (“primary transition”; Jennings et al., 2013). For this combination of reasons, we developed approaches to investigate the genetic programs responsible for initiating pancreas and liver development directly in human embryos.

## Results

### LCA-RNA-Seq on Human Embryonic Foregut Derivatives

**Figure 1:**
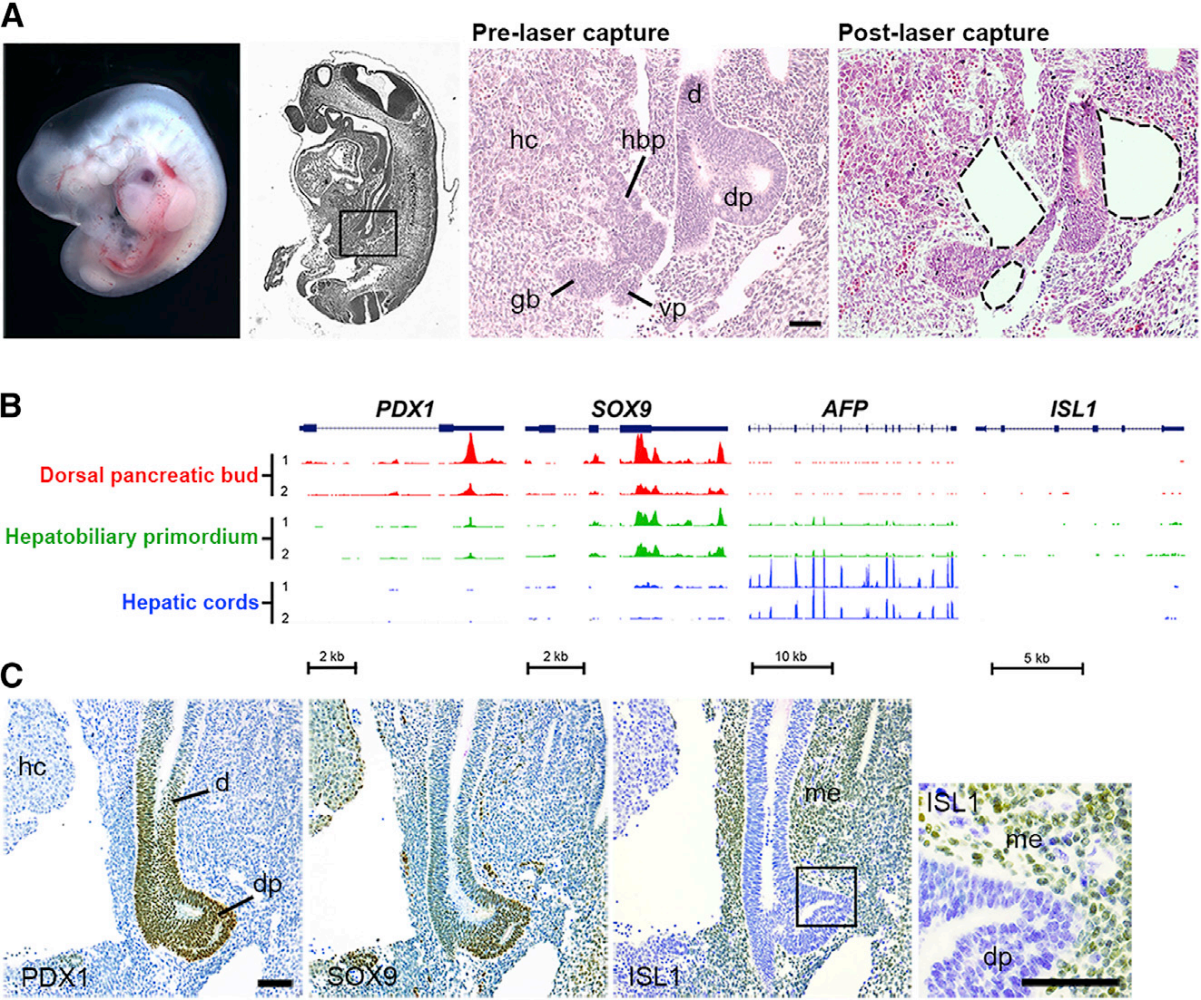
LCA-RNA-Seq of Human Foregut Derivatives (A) (Left to right) Human embryo at CS13 (30–33 days post conception [dpc]) with a sagittal section stained by H&E showing the boxed area at higher magnification before and after laser capture (LC). Post LC shows dissection of the dorsal pancreatic bud (dp), hepatobiliary primordium (hbp), and ventral pancreatic bud (vp) either side of the duodenum (d). gb, gallbladder. Insufficient total RNA was obtained from vp for LCA-RNA-seq. LC of the hepatic cords (hc) is not shown in this image. (B) Transcript profiles in two replicates (1 and 2) for key genes with known restricted expression across the dissected tissues. Lack of *ISL1* detection indicated no contamination of dp by mesenchyme (me). (C) Sagittal sections of a human embryo at CS14 (33–35 dpc) counterstained with toluidine blue following immunohistochemistry (brown) for PDX1, SOX9, and ISL1. The boxed area demonstrates positive ISL1 staining in adjacent mesenchyme. Scale bars represent 100 μm.

Human embryogenesis is categorized by Carnegie stages (CS), which extend up to 56–58 days post conception (dpc) (O'Rahilly and Muller, 2010; Table S1). At the start of the fifth week, pancreatic patterning of the distal foregut can be discerned (CS12) followed by dorsal pancreatic outgrowth from CS13 (Jennings et al., 2013). At the same time, the liver bud is comprised of the first hepatic cords connected to a hepatobiliary primordium (HBP). We identified these structures in serial tissue sections in 13 human embryos from late CS12 to early CS14 and devised methodology for laser capture (LC), RNA isolation (including DNase treatment), amplification (A), and deep sequencing (LCA-RNA-seq; Figure 1A). LC of 500,000–850,000 μm^2^ per tissue type per biological replicate (Table S1) yielded ∼15 ng total RNA for each sample. RNA amplified proportionately across tissues for genes encoding the TF, FOXA2, or “house-keeping” protein, GAPDH (Figure S1), and sequencing yielded similar mapping statistics across tissue types and replicates (Table S1). To confirm accurate dissection, we analyzed transcription of *PDX1, SOX9*, *AFP*, and *ISL1* as genes with restricted expression profiles. Both *PDX1* and *SOX9* were appropriately detected in the pancreas and to a lesser extent in the HBP (Jennings et al., 2013), while *AFP* was predominantly expressed in the hepatic cords. The effective absence of *ISL1* (in total only two reads) specifically excluded contamination of the pancreatic dataset with mesenchyme (Figures 1B and 1C). GATA4 is present in perihepatic mesenchyme but not detected in hepatoblasts or hepatocytes (Jennings et al., 2013). *GATA4* transcripts most likely indicated some heterogeneity in the hepatic cord samples. The most highly expressed genes across all samples included those encoding several key liver-specific proteins (e.g., *AFP, APOA2, SERPINA1*, and *APOB*) but also multiple imprinted genes, several of which transcribed non-coding RNAs (e.g., *H19*, *MALAT1*, and *MEG3*) (Tables S2A and S2B).

### Defining Specific Transcriptional Signatures

**Figure 2:**
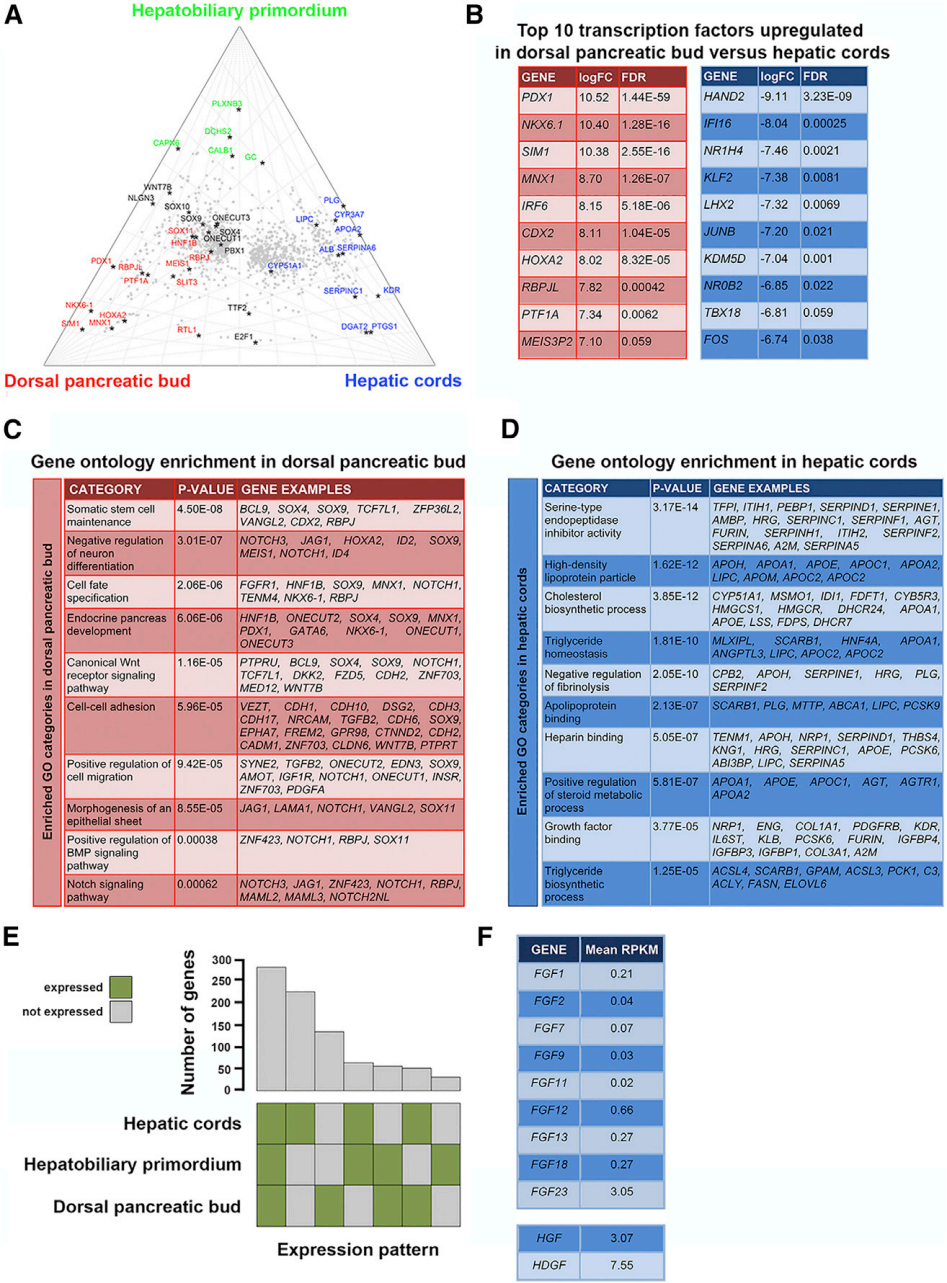
Differential Expression and Gene Ontology Enrichment Across Embryonic Foregut Outgrowths (A) Ternary log-fold plot of differential gene expression (scaled to zero at the center and a maximum of 1 at each corner) across the three tissues. Mean plot position for each gene with EdgeR false discovery rate (FDR) ≤0.01 in at least one pairwise comparison is shown as a gray dot. Selected genes are highlighted for dorsal pancreatic bud (red), hepatic cords (blue), and hepatobiliary primordium (green). (B) The ten most upregulated transcription factors (by log-fold change) in dorsal pancreatic bud (red) versus hepatic cords (blue). (C and D) Most significantly enriched GO terms for dorsal pancreatic bud (C) versus hepatic cords (D) (FDR <0.0001). (E) Bar chart and accompanying Eulergrid showing numbers of genes and the patterns of their expression across the three tissues. Green squares indicate genes expressed in either replicate (defined as >10 reads after normalization). (F) Mean RPKM for growth factors detected in the hepatic cord datasets that are relevant to hPSC differentiation protocols *in vitro*.

Based on their common origin from distal foregut, we reasoned that comparative analysis would identify the genetic programs responsible for initiating development of either the pancreas or liver. Pancreas-enriched expression of TFs, such as *NKX6-1*, *PTF1A*, and *MNX1*, concurred with their critical developmental roles in mouse (Jennings et al., 2015; Figures 2A and 2B and Tables S4A and S4C). Gene ontology (GO) analyses discovered pancreas-enriched NOTCH, BMP, and WNT signaling, similar to mouse (Rodríguez-Seguel et al., 2013) and validating the use of the relevant ligands when differentiating human pluripotent stem cells (hPSCs) to pancreatic endoderm (Rezania et al., 2014; Figure 2C; Table S4A). Despite their primitive appearance, the hepatic cords were enriched for metabolic and biosynthetic processes (Figure 2D and Tables S3A, S3B, and S4B). Regardless of threshold (reads per kilobase of transcript per million mapped reads [RPKM] ≥ 0.5, 1.0, or 10) or whether expression was detected in either or both sample replicates, exceptionally few genes emerged as characteristic of the HBP (Figures 2A and 2E); approximately 4-fold less compared with the dorsal pancreatic bud and 7-fold less compared with the hepatic cords (Figure 2E). The most distinguishing was *PLXNB3*, which encodes the receptor for Semaphorin 5A. The hepatic cord datasets sampled local growth factors relevant to *in vitro* hPSC differentiation toward hepatocytes (Figure 2F). HGF and FGF2 or -4 are widely used in current protocols. In fact, heparin-binding growth factor (*HDGF*), not *HGF*, was the most highly expressed by RPKM, while *FGF23*, not *FGF2* (barely detected) or *FGF4* (not detected), was the most highly detected FGF family member (Figure 2F).

**Figure 3:**
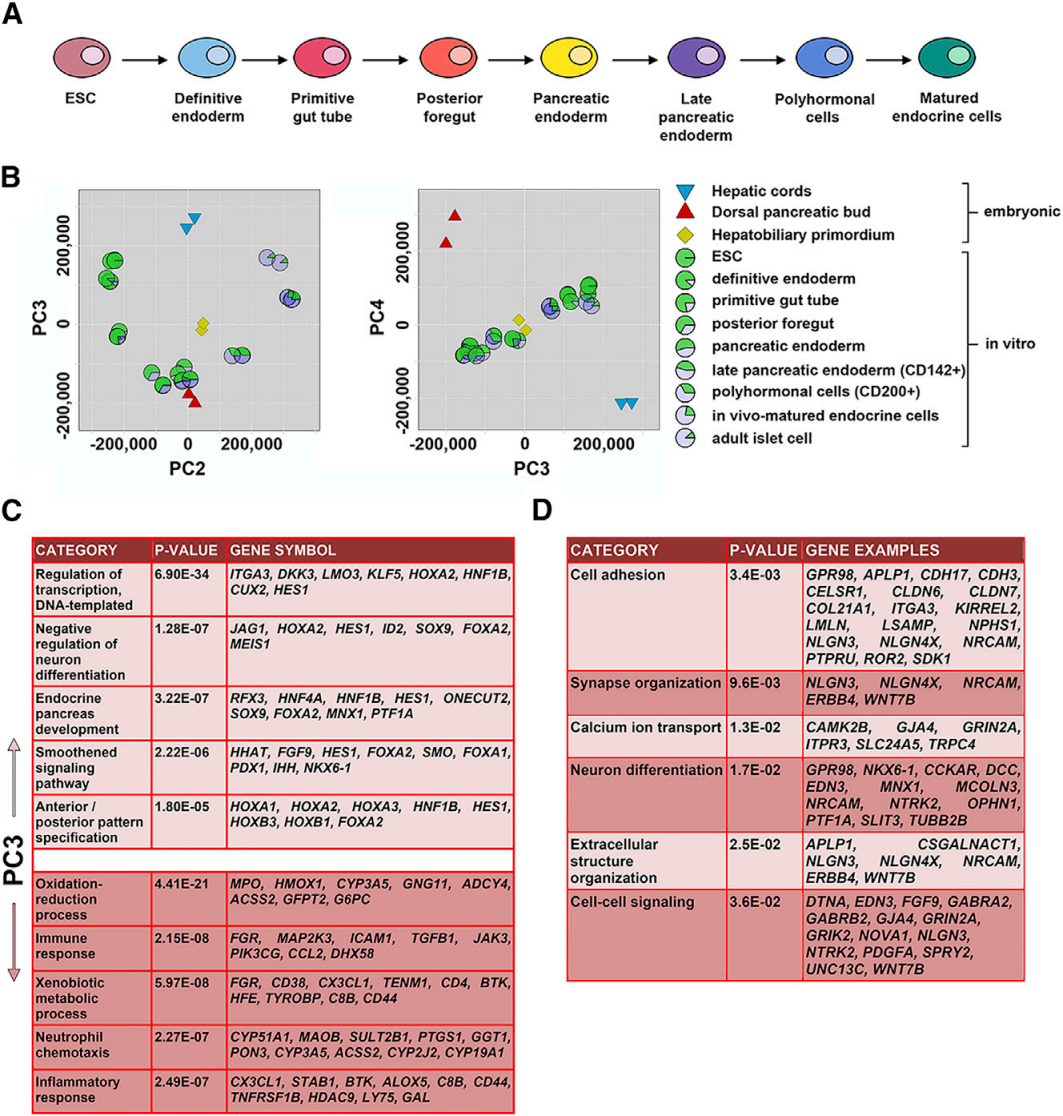
Comparison of In Vitro hPSC Differentiation with Native Human Embryonic Development (A) Schematic overview of *in vitro* hPSC differentiation toward pancreatic beta cells. (B) Principal components analysis (PCA) of rank-normalized datasets with sample loadings for PC3 plotted against those of PC2 (left) and PC4 (right). The hPSC differentiation transiently approximated to the native dorsal pancreatic bud at the stage of *in vitro* pancreatic endoderm (the lowest PC3 loadings and PC2 loadings near zero) (left). In contrast, PC4 discriminated the native from *in vitro* pancreatic cells (right). (C) The ten most enriched biological process GO terms for gene expression underlying the lowest (top panel) and highest (bottom panel) PC3 loadings, indicative of dorsal pancreas and hepatic cords, respectively. (D) Most significantly enriched biological process GO terms for gene expression (≥10 reads in both replicates) underlying low PC3 and high PC4 loadings (<−0.005 and >0.005), which discriminated native dorsal pancreatic bud from PSC-derived pancreatic endoderm.

Previously, we have scrutinized the fidelity of pancreatic differentiation from hPSCs against human pancreas from later development, after branching, benchmarked mostly against non-endodermal lineages and without consideration of the hPSC differentiation time course (Cebola et al., 2015). To analyze differentiation dynamically against earlier pancreatic development and including closely related endoderm derivatives, we normalized and integrated data from sequentially differentiated hPSCs (Figure 3A; Xie et al., 2013) and undertook principal components (PC) analysis (PCA; Table S5). Variance in PC1 reflected the different sample sources and was uninformative (Figure S2). In contrast, PC2 loadings close to zero identified distal foregut outgrowths. Plotting PC3 against PC2 depicted differentiation from hPSCs to pancreatic endocrine cells as a U-shape with unique transient approximation to the native pancreatic bud at the appropriate stage of *in vitro* pancreatic endoderm (Figure 3B). Underlying this proximity was expression of key pancreatic genes such as *ONECUT2*, *MNX1*, and *SOX9* as part of the associated GO term “endocrine pancreas development” (Figure 3C; Tables S6 and S7A). In contrast, plotting PC4 and PC3 emphasized the relative deficiency of the hPSC-derived pancreatic endoderm in gene expression underlying GO terms for “cell adhesion” and “cell-cell signaling” and expression of the key pancreatic genes *NKX6-1* and *PTF1A* (Figure 3D).

### Distinct Expression Marks the Human Dorsal and Ventral Pancreatic Buds

**Figure 4:**
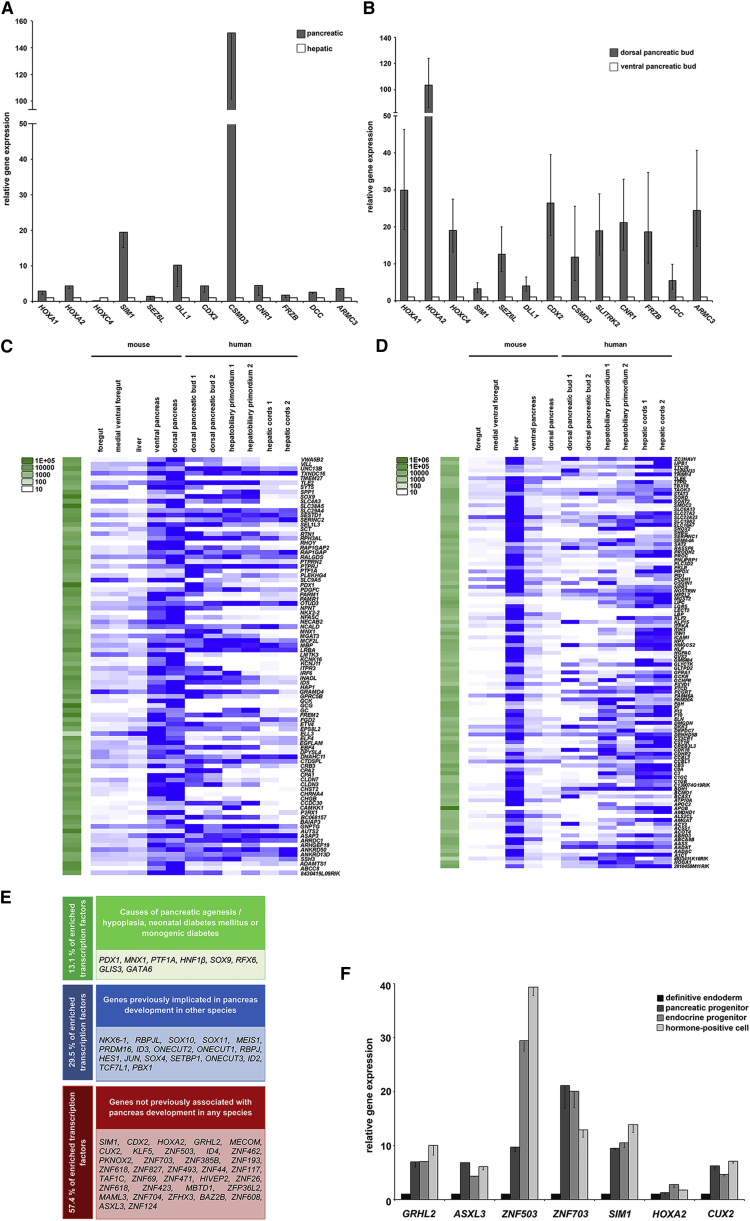
Distinct Expression Patterns Mark Human Dorsal and Ventral Pancreatic Buds and Transcription Factor Enrichment Identifies Clinically Important Pancreatic Regulators (A) Quantitative (q) RT-PCR in hPSC-derived pancreatic endoderm (pancreatic) and hepatic differentiation (hepatic) for genes identified from low PC3 loadings and absent expression in hepatic cords and HBP. Detection was normalized to *ACTB.* Error bars, ± SEM (n = 3). (B) qRT-PCR for the same genes in dorsal and ventral pancreatic buds normalized to *ACTB* and *PDX1.* Error bars, min/max fold difference. (C and D) Heatmap of transcripts that showed significant differential expression between dorsal pancreatic bud (C) and liver bud (D) in mouse (Rodríguez-Seguel et al., 2013) integrated with the human LCA-RNA-seq data. The blue to white scale represents high to low expression. Read counts were quantile normalized and scaled to the maximum value across all samples (shown in green on the left). (E) Genes encoding transcription factors enriched in the dorsal pancreatic bud compared with hepatic cords (fold change > 2, FDR <0.001) and categorized as either known (green and blue) or unrecognized (red). (F) Pancreatic transcription factor gene expression by qRT-PCR during *in vitro* pancreatic differentiation of human PSCs. Expression levels are shown relative to the definitive endoderm (DE) stage. Error bars represent ± SEM (n = 3).

PC3 loadings in Figure 3B clearly separated the dorsal pancreatic bud from either HPB or hepatic cords with the GO term for anterior/posterior pattern specification (Figure 3C; Table S6). This led us to hypothesize that patterning genes and others with extreme low PC3 loadings and absent expression in either hepatic cords or HBP (Table S2A) might discriminate ventral from dorsal foregut structures. We identified 13 genes (*HOXA1*, *HOXA2*, *HOXC4*, *SIM1*, *SEZ6L*, *DLL1*, *CDX2*, *CSMD3*, *SLITRK2*, *CNR1*, *FRZB*, *DCC*, and *ARMC3*). Consistent with our LC data, expression of many of these genes was enriched in hPSCs differentiated to pancreatic endoderm compared with hepatoblasts/early hepatocytes (Figure 4A). Although exceptionally limited RNA from the tiny ventral pancreatic bud precluded LCA-RNA-seq, we isolated sufficient from four embryos (Table S1) to question whether these 13 genes might also discriminate the dorsal from the ventral pancreatic bud. By quantitative (q) RT-PCR, *PDX1* was at least as well expressed in the ventral as in the dorsal bud and used to normalize the data alongside *ACTB.* All 13 of our genes were enriched in the dorsal compared with the ventral pancreatic bud (Figure 4B). Taken together with the PC3 loadings, these data imply that hPSCs differentiate down a dorsal rather than a ventral pancreatic program.

### Comparison of Early Pancreas and Liver Development between Human and Mouse

We have previously shown molecular differences between very early human and mouse pancreas (Jennings et al., 2013), prompting us to interrogate comparable mouse transcriptomes from E10.5 (Rodríguez-Seguel et al., 2013). We identified those genes in mouse with enrichment in dorsal pancreas compared with the liver bud (Figure 4C). Approximately half of the genes also showed clear enrichment in human dorsal pancreatic bud compared with the hepatic cords. However, a number of genes failed to show such differential expression (e.g., *TXNDC16*, *SLC29A4*, or *CHST2*, alongside *NKX2-2* as expected; Jennings et al., 2013) or were more enriched in human liver (e.g., *DNAHC11*, *LPAR1*, or *ASAP3*). In contrast, most genes upregulated in the mouse liver bud compared with dorsal pancreas were similarly upregulated in human hepatic cords (Figure 4D).

### Transcription Factor Codes Regulating Human Pancreas and Liver Buds

We scrutinized TF codes in liver and pancreas buds in two ways. Firstly, we cataloged all TFs in each dataset by expression level (Table S7A). There were 655 TFs in the pancreatic bud, 574 in the HBP, and 637 in the hepatic cords. From this repertoire, we identified those TFs imputed to regulate the most differentially expressed genes based on known binding events or motif discovery (Janky et al., 2014). SOX9 (38% of the top 1,000 pancreas-enriched genes) and RORA (44%) were assigned as key regulators of the pancreatic program, while approximately half of the most liver-enriched genes were ascribed regulation by FOXA family members, the transcriptional coactivator EP300 and HNF4A (Table S7B). In addition, to capture the wider repertoire of key pancreatic TFs, we filtered differential expression between pancreas and liver based on fold change and significance. This yielded 61 TFs, including virtually all causes of human pancreatic agenesis or hypoplasia such as *PDX1*, *PTF1A*, and *RFX6* (Jennings et al., 2015; Figure 4E). A further 30% are known to function in pancreas development in other species (e.g., *NKX6-1* and *ONECUT1*; Jennings et al., 2015)*.* Over half (57.4%) were previously not associated with pancreas development, including many zinc finger TFs (Figure 4E). qRT-PCR for a subset of these factors showed increased expression as hPSCs underwent pancreatic endoderm differentiation (Figure 4F).

## Discussion

We have deciphered the transcriptomic programs regulating the earliest development of the pancreas, liver, and biliary tree in human embryos.

The HBP transcriptome was relatively undistinctive. *PLXNB3*, the most characteristic HBP gene, is among seven genes on the X chromosome that can be deleted as part of the Contiguous ABCD1/DXS1375E Deletion Syndrome (CADDS; OMIM #300475), which includes severe cholestatic jaundice due to obstructed bile flow (Iwasa et al., 2013). None of the other six genes were enriched in the HBP, offering PLXNB3 as a plausible regulator of human bile duct development.

Comprehensive transcriptomic signatures were discerned for hepatic cords and pancreatic bud. The imputed regulatory role for SOX9 fits with the pancreatic hypoplasia observed in campomelic dysplasia (Piper et al., 2002) and the pancreatic enrichment of WNT, NOTCH, and BMP signaling pathways, which are known to regulate *SOX9* (Figure 2C; Pritchett et al., 2011). The value of our data will increase as computational tools, such as iRegulon, add binding information for additional TFs. Our 35 pancreatic TFs (Figure 4E) include some that are already networked, such as ZNF503 and ZNF703 as targets of HOXA2 in the branchial arches (Amin et al., 2015). In addition, genes encoding 15 of the 35 TFs (*SIM1*, *CDX2*, *GRHL2*, *CUX2*, *KLF5*, *ZNF503*, *ID4*, *ZNF462*, *ZNF703*, *ZFP36L2*, *MAML3*, *ZNF704*, *ZFHX3*, *ZNF608*, and *ASXL3*) are associated with *cis*-regulatory modules controlled by PDX1, GATA6, FOXA2, HNF1B, and/or ONECUT1 in hPSC-derived pancreatic endoderm (Cebola et al., 2015).

In liver, current protocols for generating hepatocyte-like cells from hPSCs result in immature phenotypes (Baxter et al., 2015) implying added factors may not mirror those from human development. Here, we have demonstrated HDGF and FGF23 as two factors from human embryogenesis for exploration in hPSC protocols. As an alternative to hPSC differentiation, imputing HNF4A and FOXA factors as central to the molecular circuitry in the developing liver concords with their ability to reprogram human fibroblasts toward hepatocytes (Huang et al., 2014).

In summary, LCA-RNA-seq has decoded the transcriptomic programs operational at the inception of human pancreas and liver development. The data are anticipated to help refine hPSC differentiation protocols, prioritize factors for programming cell fate, including the potential interconversion of hepatic and pancreatic cell fate by transdifferentiation, and understand developmental disorders in patients. Alongside other studies in later stage embryos (Cebola et al., 2015, Gerrard et al., 2016), the data pave the way for a comprehensive genomic atlas of human organogenesis.

## Experimental Procedures

### Human Tissue, Immunohistochemistry, and Microdissection

The collection, use, and storage of human embryos (Table S1) from social termination of pregnancy was carried out as previously (Jennings et al., 2013) with ethical approval from the North West Research Ethics Committee under the Codes of Practice of the UK Human Tissue Authority. In brief, human embryos were fixed within 1 hr in 4% paraformaldehyde (PFA) under RNase-free conditions, processed, and embedded in paraffin wax for orientated sectioning in either the transverse or sagittal planes at 5 μm intervals. Landmark H&E staining was undertaken every eighth section. Tracing the correct structures for LC was achieved by immunostaining occasional sections (Supplemental Experimental Procedures; Figure 1C). Intervening hematoxylin-stained sections were then microdissected under ×40 objective magnification using a PALM Microbeam 3 system (Carl Zeiss GmbH, Germany). Material was catapulted into a 500 μL AdhesiveCap (Carl Zeiss GmbH, Germany) and stored at −80°C.

### RNA Extraction, Amplification, and PCR

Total RNA was extracted using the RNeasy FFPE kit (QIAGEN, Manchester, UK) according to the manufacturer's instructions, including DNase treatment. RNA integrity (RNA integrity number ≥ 7) and quantity were determined using an Agilent 2,100 Bioanalyzer (Agilent Technologies, Palo Alto, CA, USA). Total RNA (15 ng) was amplified and converted to cDNA using the Ovation RNA-seq system V2 (NuGEN Technologies, San Carlos, CA, USA; Dafforn et al., 2004). To assess uniformity of amplification, qPCR was performed on samples post amplification (Applied Biosystems, Life Technologies) and compared with qPCR following RT of unamplified (control) samples (Figure S1). RT and qPCR were performed as described previously (Baxter et al., 2015; primers listed in Supplemental Experimental Procedures). Severe material limitation led to a single analysis of gene expression in the ventral pancreatic bud.

### Deep Sequencing, Mapping, Quantification, and Differential Expression

Paired-end sequencing (50 bp) was carried out using an Illumina HiSeq 2000 at the Wellcome Trust Centre for Human Genetics, Oxford, UK. Reads were mapped to the GENCODE 15 transcriptome (Harrow et al., 2012) using TopHat version 1.4.1 (Trapnell et al., 2009). Gene-level transcript abundance (read counts and RPKM) was estimated by an algorithm implemented in the Partek Genomics Suite (version 6.6 [6.12.1227]; Partek Inc., St. Louis, MO, USA) (Tables S2A and S2B). After filtering mitochondrial genes, ribosomal RNAs, and two other multi-locality RNAs (Metazoa_SRP and 7SK) the number of mapped reads varied from zero (for >50% of genes) to >90,000 (e.g., *APOB* in liver). Differential expression was examined in the R/Bioconductor package EdgeR (version 3.0.8; Robinson et al., 2010) using a generalized linear model (count = tissue + replicate) and the default trimmed mean of M values (TMM) scaled differences in library size (McCarthy et al., 2012). For comparison with pancreatic hPSC differentiation (Xie et al., 2013), RNA-seq data were retrieved from ArrayExpress, remapped, and quantified as above. PCA was performed on the combined rank-normalized gene-level abundances from both datasets. The mouse RNA-seq dataset (GEO: GSE40823) (Rodríguez-Seguel et al., 2013) was downloaded and remapped to the mm10 genome using STAR (version 2.4.2a; Dobin et al., 2013) with gene-level read counts calculated according to the GENCODE M5 annotation. Human and mouse read counts were combined by biomaRt (Durinck et al., 2009) using gene i.d. mappings from Ensembl and quantile normalized. Genes were filtered for one-to-one orthologs.

### Gene Set Enrichment

Sets of genes enriched between the different LCA-RNA-seq datasets were assessed for GO term enrichment with EdgeR false discovery rates <10^−4^. Fisher's exact test was applied with the elimination algorithm as implemented in the topGO R package (version 2.12.0). Additional data and annotations were obtained from other Bioconductor R packages (org.Hs.eg.db [2.9.0], GO.db [2.9.0], AnnotationDbi [1.22.6]).

Gene-level PCA projected loadings were used to test for GO gene set enrichment employing one-sided Wilcoxon rank-sum tests to test separately for enrichment at both ends of the loading distributions. This was implemented within the topGO framework and used the elimination algorithm to traverse the GO ontologies.

### Analysis of Regulation by Transcription Factors

The 1,000 genes most differentially expressed in dorsal pancreas (logFC > 0) or hepatic cords (logFC < 0) were loaded into Cytoscape (version 3.2.1.) and used as queries to the iRegulon plug-in (version 1.3, build 1024) (Janky et al., 2014). The default iRegulon parameters search for enrichment of either known motifs or experimental TF binding data within 10 kb of the transcription start sites. Pancreatic analysis was constrained to motif discovery to overcome the relative lack of pancreatic binding data in iRegulon compared with data for hepatocytes. The putative regulators returned from the iRegulon analysis were filtered according to expression in the LCA-RNA-seq datasets (Table S7B).

### hPSC Differentiation

*In vitro* differentiation of hPSCs to pancreatic endoderm (H9) and hepatoblast-early hepatocyte-like cells (HUES7) was undertaken as reported previously (Baxter et al., 2015, Cebola et al., 2015) with RT-qPCR at 3, 13, 16, and 27 days (pancreatic protocol) and 3, 10, 15, and 30 days (hepatic).

## Author Contributions

R.E.J. researched data and co-wrote the manuscript with N.A.H. D.T.G. and A.A.B. researched data and drafted sections of the manuscript. K.P.H. and N.B. edited the manuscript and contributed discussion. L.V., M.C., S.W., J.S., S.C., and S.J.W. researched data and contributed discussion. N.A.H. is the guarantor for human material collection and oversaw the project. A.A.B. and D.T.G. contributed equally.

## Accession Numbers

RNA-seq datasets have been deposited into ArrayExpress: E-MTAB-3259 and are freely available via http://www.humandevelopmentalbiology.manchester.ac.uk/

## Supplemental Information

Document S1. Supplemental Experimental Procedures, Figures S1 and S2

Table S1. Carnegie Stages of Human Development and LCA-RNA-Seq Mapping Summary

Table S2. Gene-Level Abundance: Read Count and RPKM

Table S3. Differential Gene Expression: Dorsal Pancreatic Bud versus Hepatic Cords; Hepatic Cords versus HBP; Dorsal Pancreatic Bud versus HBP

Table S4. Gene Ontology Enrichment of Upregulated Genes in Dorsal Pancreatic Bud and Hepatic Cords

Table S5. Comparison between Human Embryonic Datasets and *In Vitro* Differentiated PSCs by Principal Components Analysis

Table S6. Gene Ontology Terms and the Genes Underlying Them from the Extremes of PC3

Table S7. Human Embryonic Transcription Factor Assembly and Transcriptional Regulatory Networks of Dorsal Pancreatic Bud and Hepatic Cords

Document S2. Article plus Supplemental Information
