# Supplementary material for: Laser Capture and Deep Sequencing Reveals the Transcriptomic Programmes Regulating the Onset of Pancreas and Liver Differentiation in Human Embryos
Source: Stem Cell Reports. 2017 Oct 19;9(5):1387–94. doi: 10.1016/j.stemcr.2017.09.018 (PMC5830993; doi:10.1016/j.stemcr.2017.09.018)
Supplement: Document S1. Supplemental Experimental Procedures, Figures S1 and S2 [file mmc1.pdf]

Stem Cell Reports, Volume *9*

Supplemental Information

Laser Capture and Deep Sequencing Reveals the Transcriptomic Programmes Regulating the Onset of Pancreas and Liver Differentiation in Human Embryos

Rachel E. Jennings, Andrew A. Berry, David T. Gerrard, Stephen J. Wearne, James Strutt, Sarah Withey, Mariya Chhatriwala, Karen Piper Hanley, Ludovic Vallier, Nicoletta Bobola, and Neil A. Hanley

Figure S1

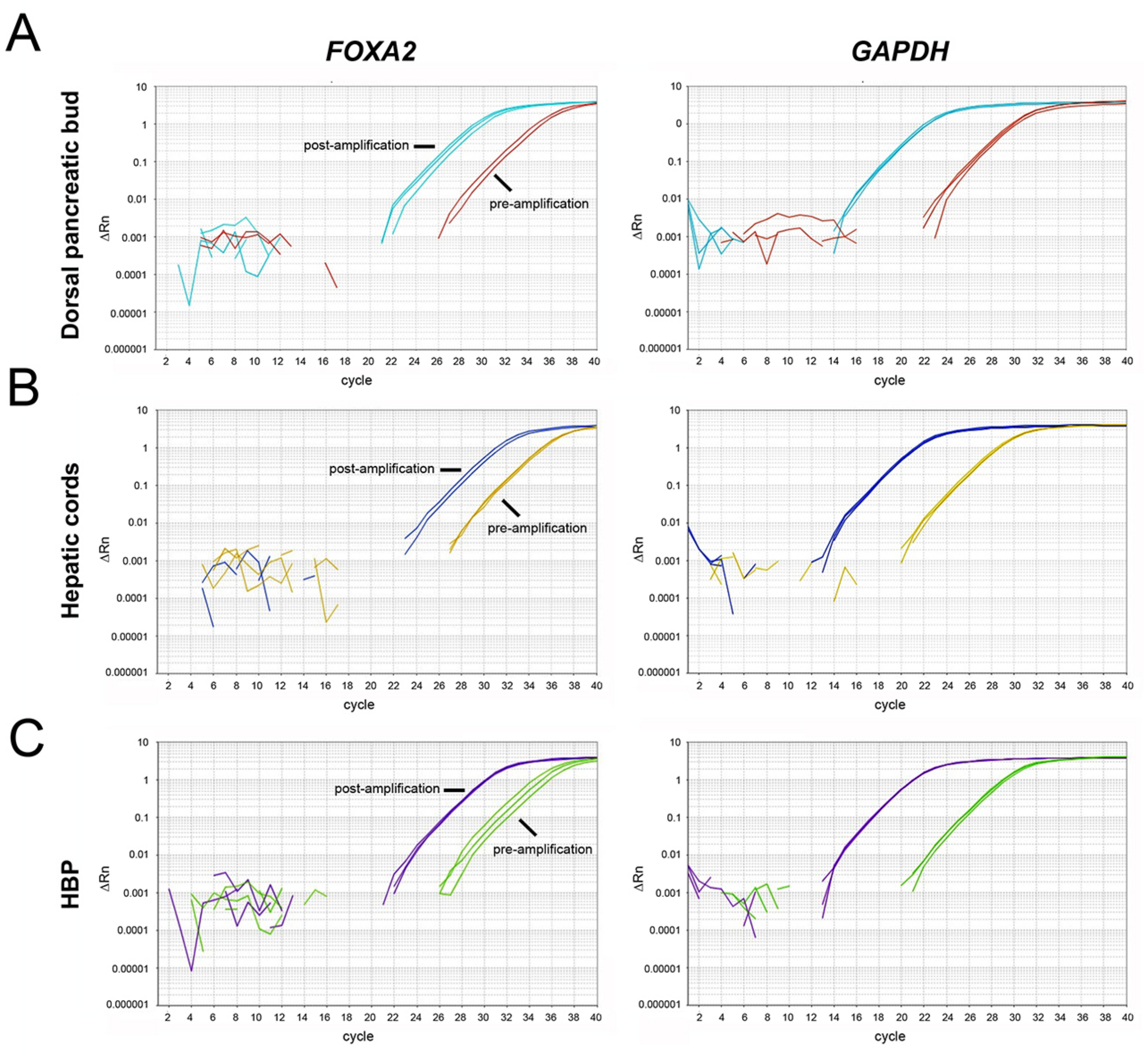

Figure S2

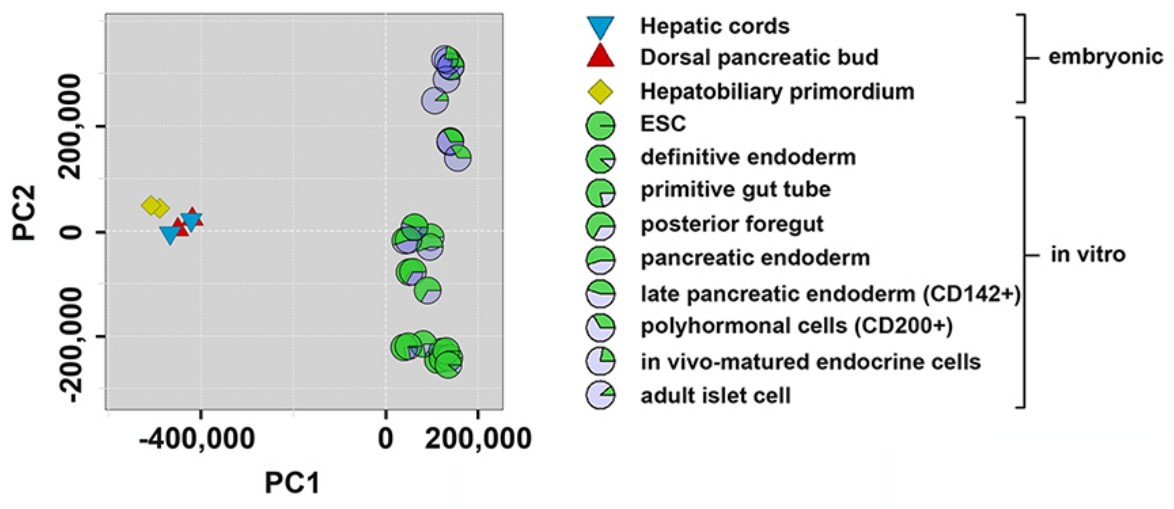

Supplemental figure legends

Figure S1. Validation of amplification as part of LCA-RNAseq. Related to Figure 1.

(A-C) RT-qPCR amplification plots pre-and post-amplification for *FOXA2* and *GAPDH* in dorsal pancreatic bud (A), hepatic cords (B) and HBP (C) illustrate consistent amplification across samples. The plots show PCR cycle number (*x* axis) plotted versus log of change (*y* axis).

Figure S2. Principal components (PC) analysis of human embryonic datasets and in vitro differentiated PSC cells (Xie et al. 2013). Related to Figure 3.

PC1 is plotted versus PC2 and illustrates that variance in the first PC is related to sample source (i.e. laser captured fixed tissue versus PSC derivatives or adult islet cells). PCs 2-4 are shown in Fig. 3 of the main text.

Supplemental table legends

Table S1. (A) Carnegie Stages of Human Development; (B) LCA-RNAseq mapping summary

(A) Timeline of human embryonic development (Carnegie stages and days post-conception). Adapted from Jennings et al. 2015. (B) LCA-RNAseq mapping summary

Table S2. (A) Gene level abundance: read count; (B) Gene level abundance: RPKM

(A) Raw read counts for each LCA-RNAseq sample with annotations from GENCODE15. Read counts are non-integers due to the isoform level counting algorithm used in Partek Genomics Suite (version 6.6 [6.12.1227]; Partek Inc., St. Louis, MO, USA) (Xing et al., 2006). (B) Reads per kilobase per million mapped reads (RPKM) with annotations from GENCODE15 estimated by algorithm implemented in Partek Genomics Suite (version 6.6 [6.12.1227]; Partek Inc., St. Louis, MO, USA) (Xing et al., 2006).

Table S3. (A) Differential gene expression: dorsal pancreatic bud v hepatic cords; (B) Differential gene expression: hepatic cords v HBP; (C) Differential gene expression: dorsal pancreatic bud v HBP

Gene-wise differential expression test results using EdgeR. (FDR - False discovery rate). Log-fold change is positive for genes enriched in dorsal pancreatic bud (versus hepatic cords) and negative for genes enriched in hepatic cords (versus dorsal pancreatic bud) (A); (B) log-fold change is positive for genes enriched in hepatic cords (versus HBP) and negative for genes enriched in HBP (versus hepatic cords); (C) log-fold change is positive for genes enriched in dorsal pancreatic bud (versus HBP) and negative for genes enriched in HBP (versus dorsal pancreatic bud).

Table S4. (A) Gene ontology enrichment of upregulated genes in dorsal pancreatic bud; (B) Gene ontology enrichment of upregulated genes in hepatic cords

Top enriched gene ontology (GO) terms amongst the set of genes up-regulated (FDR <0.0001) in dorsal pancreatic bud versus hepatic cords (A) and hepatic cords versus dorsal pancreatic bud (B). The fisher test was applied sequentially using the 'elimination' algorithm ('elimFisher') to reduce multiple testing, implemented in the topGO package. Each ontology (BP, CC, MF) was tested separately and results are combined here (results <0.001 shown).

Table S5. Comparison between human embryonic datasets and in vitro differentiated PSC cells (Xie et al. 2013) by principal components analysis

Combined read counts from LCA-RNAseq samples and RNAseq data from Xie et al (2013). PC1 to PC10 are principal components loading vectors for all genes and are used to produce Fig. 3B and perform subsequent GO analysis.

Table S6. Gene Ontology (GO) terms and the genes underlying them from the extremes of PC3

Most enriched GO terms amongst genes with high and low loadings on PC3. The Wilcoxon test was applied sequentially using the 'elimination' algorithm ('elimWilcoxGreater' for high loadings and 'elimWilcoxLesser' for low loadings) to reduce multiple testing, implemented in the topGO package. Each ontology (BP, CC, MF) was tested separately and results are combined here (results <0.0001 shown).

Table S7. (A) Human Embryonic Transcription Factor Assembly; (B) Transcriptional regulatory networks of dorsal pancreatic bud and hepatic cords

(A) Transcription factors were identified by KEGG, the Genecard database and GO annotations. Complete assemblies of transcription factors are shown for each cell-type where the mean standardized read count is >10. (B) The 1,000 genes most differentially expressed in dorsal pancreas (logFC >0) or hepatic cords (logFC <0) were loaded into Cytoscape (version 3.2.1.) and used as queries to the iRegulon plug-in, to decipher transcriptional regulatory networks in sets of co-expressed genes (version 1.3, build 1024; Janky et al. 2014). Each dataset was filtered for transcription factors annotated on KEGG (http://www.genome.jp/kegg-bin/get_htext?hsa03000) based on a mean count of >10 reads across the replicates.

Supplemental experimental procedures

Antibodies used in this study

Antigen	Raised in	Dilution	I.D. number	Source
PDX1	Guinea pig	1 in 500	ab47308	Abcam
SOX9	Rabbit	1 in 2000	AB5535	Millipore
AFP	Mouse	1 in 400	MAB1368	R+D systems
ISL1	Mouse	1 in 2000	39.4D5	Developmental Studies Hybridoma Bank (DSHB)

RT-qPCR primers used in this study

Gene	Forward primer	Reverse primer
CUX2	CTACGGCGACTCCAGAAGGA	AAATTCCCGGCGGAGTTCAA
PDX1	AAAGGCCAGTGGGCAGGCGG	GCGCGGCCGTGAGATGTACT
HOXA1	GTGGGCTCGCCTCAATACAT	CGTACTCTCCAACTTTCCCTGT
HOXA2	CAGACCATTCCCAGCCTGAAC	CGGCGATTTCCAGGGATTCTTTG
HOXC4	CATGGCCAGAGGGTTGGAAATTA	CGGACTGTGTTCAGGGATGTA
SEZ6L	CCGGTTTTGTGCTTGAAGGG	GCTGCCGCTTCTTCTAAAGC
SIM1	ATACATTCACCCGGCAGACC	CGCTCGATCTCATACTCCTGC
FOXA2	GAAGATGGAAGGGCACGAGC	GTACGTGTTCATGCCGTTCA
DLL1	ACCTCGCAACAGAAAACCCA	GTGTTCGTCACACACGAAGC
CDX2	GCAGCCAAGTGAAAACCAGG	CCTCCGGATGGTGATGTAGC
CSMD3	GCAAGCGAAGATGCGCTAAA	ATAAATCCTTTCACACAAGACACCG
SLITRK2	AGATCAGAACCCAGGTTGGAGG	TTCCCTTCCTTGCAAGTGGCT
CNR1	GATGCGAAGGGATTGCCCC	GATGGTGCGGAAGGTGGTAT
FRZB	GCAAGCAGTGAACGCTGTAAA	CCTCCACTACTGCAGTCACA
DCC	GAGCAGGAAGAAGTCAGTCAGT	CTGTGGGGATGGTTCTGCTT
ARMC3	TCTTACGTTTCTCCACCTTCATCT	TCCAAATCCAGCACTAAAATGATAA
GRHL2	CAAAGCAAGTGACAGCCAAG	CTTTGTTGAGGTAGGTCATGG
ASXL3	GTGGAATCCCCCACGTCATC	TTTTTCTAGTGCCAGGCGGG
ZNF503	GCACGAACTGGCCACATTTT	GGGCTCTTCTTGGCATCGAG
ZNF703	CCATTGAGCTGGACGCCAAGA	CCGCCACCGAGTTGAGTTT
GAPDH	CGACCACTTTGTCAAGCTCA	GGGTCTTACTCCTTGGAGGC
BETA-ACTIN	CCAACCGCGAGAAGATGA	CCAGAGGCGTACAGGGATAG
